# Corrosion mitigation in desalination plants by ammonium-based ionic liquid

**DOI:** 10.1038/s41598-021-00925-z

**Published:** 2021-11-02

**Authors:** M. A. Deyab, Q. Mohsen

**Affiliations:** 1 Egyptian Petroleum Research Institute (EPRI), Nasr City, Cairo, Egypt; 2 Department of Chemistry, College of Sciences, Taif University, Taif, Saudi Arabia

**Keywords:** Chemistry, Corrosion

## Abstract

CuNi (90:10) alloy is widely used in desalination plants. CuNi alloy corrosion in sulfide-containing seawater is the fundamental problem in the desalination industry. Here we have confronted this difficulty by using ammonium-based ionic liquid (Diethyl (2-methoxyethyl)-methyl ammonium Bis(fluorosulfonyl)imide) [DEMEMA][FSI]. The results revealed that the [DEMEMA][FSI] can suppress Cu–Ni alloy corrosion in a solution of (3.5% NaCl + 10 ppm sulphide) with an efficiency of 98.4% at 120 ppm. This has been estimated by electrochemistry and gravimetry. Furthermore, [DEMEMA][FSI] inhibits the growth of sulfate-reducing bacteria SRB in saline water. Surface morphology testing confirmed [DEMEMA][FSI] adsorption on Cu–Ni surface alloys. In addition, quantum calculations have been used to theoretically predict inhibition efficiency [DEMEMA][FSI].

## Introduction

Desalination from sea water is one of our time's main challenges^1,2^. The main task of desalination plants is to remove salts from marine water to produce drinking water^3^. During the desalination processes, the saline water pass though various tubes and units contain different metal and alloys^4^. The CuNi (90:10) alloy is the best alloy for constructing condensers and heat exchangers in desalination plants^5,6^. The corrosion resistance of CuNi (90:10) is very high due to the formation Cu_2_O passive film on the alloy surface^7,8^. However, this passive film can be eliminated by sulfide containing seawater^9^. Where the sulfide ions convert the strong Cu_2_O passive film to a tenuous black layer^10^. The source of the presence of sulfide ions in the seawater comes from industrial waste and/or bacteriological processes (Sulfate reducing bacteria SRB)^11^. This condition causes significant corrosion issues in desalination plant units and cooling systems^12,13^.

Organic inhibitors, such as azoles, Schiff bases and amino acids are widely used to protect CuNi alloy from corrosion^14–16^. However, these organic inhibitors are hazardous materials^17^.

Alternatively, toxic corrosion inhibitors are replaced with environmentally benign materials such as ionic liquids (ILs)^18–20^. ILs have recently been successfully used in different environments as inhibitors of corrosion^21–23^. Most of the ILs contain organic cation and inorganic anions^24^. They also have very low toxicity and low cost^25^.

Guo et al. produced ureido substituted imidazolium bromides and investigated their anti-corrosion effectiveness on steel in HCl solution^26^. They show that the imidazolium inhibitors are effective mixed-type corrosion inhibitors, with higher inhibition efficiencies as concentration and alkyl chain length increase. The inhibition mechanism of three quaternary-ammonium-derived ionic liquids was clearly discussed by Olivares-Xometl et al.^27^. The inhibition efficiency in this study ranged from 55 to 80 percent. Likhanova et al.^28^ looked into the impact of organic anions on ionic liquids as inhibitors for steel corrosion in sulfuric acid solution. They found that the ethyl sulphate anion was found to have better inhibitory properties of corrosion. Shetty et al.^29^ investigated the use of an environmentally friendly benzimidazolium-based ionic liquid as an inhibitor for aluminum alloy corrosion in acidic solution. The inhibition efficiency of ionic fluid based on benzimidazium was 98.7% in Hydrochloric acid and 98.8% in sulfuric solutions.

In this work, for the first time, ammonium based ionic liquid (Diethyl (2-methoxyethyl)-methyl ammonium Bis(fluorosulfonyl)imide) [DEMEMA][FSI] was used to protect CuNi alloy from corrosion in sulfide containing seawater medium. Our strategy here depends on the decrease of the corrosive action of seawater and the inhibition of the production of sulfide ions from biological activities of SRB.

## Materials and methods

CuNi (90:10) alloy was obtained from desalination unit in Egypt with composition: Ni = 10%, Fe = 1.2%, Mn = 0.8%, Cu = Remaining. The preparation of alloy surface before the experiments was conducted according to ASTM G1—03(2017)e1^30^.

Ammonium based ionic liquid (Diethyl (2-methoxyethyl)-methyl ammonium Bis(fluorosulfonyl)imide) [DEMEMA][FSI] (purity 98%) was purchased from Sigma-Aldrich.

The analar sodium chloride and sodium sulfide with distilled water were used to prepare the corrosive solutions.

All the electrochemical tests were conducted in a standard cell fitted with three-electrode (CuNi alloy, Pt, Ag/AgCl) and recorded by Gamry 3000 electrochemical workstation. The polarization experiments conditions are scan rate = 1.0 mV s^−1^, potential range =  ± 250 mV vs. OCP, solution temperature = 298 K. Electrochemical impedance measurements (EIS) experiments conditions are frequency range = 30 kHz–0.01 Hz and amplitude = 10 mV at OCP.

Weighing the cleaned CuNi alloy electrodes (dimension 1.5 × 2.0 × 0.2 cm) before and after immersion in tested solutions for 48 h at 298 K was used to calculate gravimetric analysis. The following formula is used to calculate the corrosion rate (*C*_R_):

1 $$C_{{\text{R}}} = \frac{W}{St}$$

*(W* = mass loss (mg), *S* = electrode surface area (cm^2^), *t* = immersion time (h)).

Desulfovibrio desulfuricans (SRB stains) was isolated from EAST BAHARIA oil fields (Egypt). Postgate’s C (PGC) medium was used to inoculate SRB cultures. After 30 min of purging with high-purity nitrogen, the medium was degassed and autoclaved at 120 °C. Seven-day-old bacteria were injected into the testing system. Total SRB count (CFU) was calculated according to ASTM D4455-85^31^.

The scanning electron microscopy (SEM) and energy dispersive X-ray spectroscopy (EDX) was used for surface characterization (model: JEOL /JSM6510) equipped with EDX unit.

VAMP module (materials-studio, Accelrys) was used to calculate the quantum chemical parameters for [DEMEMA][FSI] in gas phase.

## Results and discussion

### Electrochemical measurements

**Figure 1:**
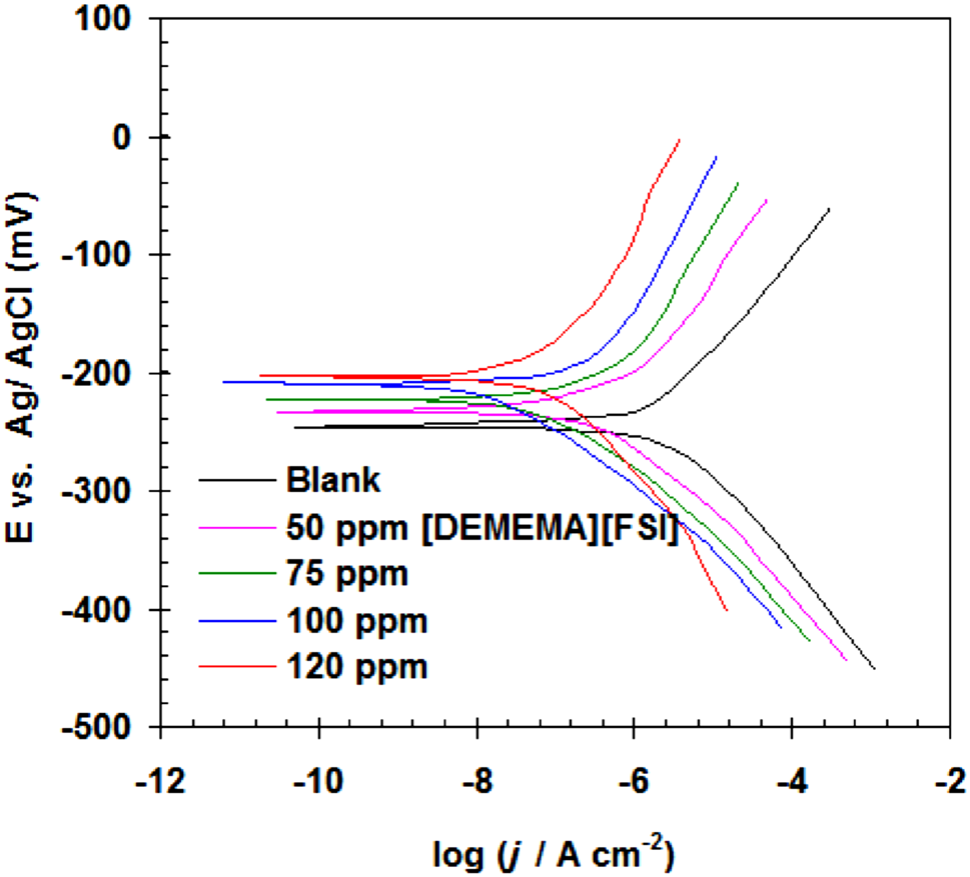
Polarization curves of Cu–Ni alloy in (3.5% NaCl + 10 ppm sulfide) solution in the absence and presence of [DEMEMA][FSI] at 298 K.

The impacts of [DEMEMA][FSI] on the corrosion rate of Cu–Ni alloy in (3.5% NaCl + 10 ppm sulfide) solution was analyzed using polarization tests (see Fig. 1). As evident from Fig. 1, the Tafel lines were shifted to the low current density by adding [DEMEMA][FSI]. The shifting extent of the Tafel lines depends on the concentration of [DEMEMA][FSI]. Tafel lines in Fig. 1 were used to extract the corrosion current density (*i*_corr_), corrosion potential (*E*_corr_) and anodic/cathodic Tafel slopes (*B*_a_, *B*_c)_ (see Table 1)^32^. The *i*_corr_ of Cu–Ni alloy in (3.5% NaCl + 10 ppm sulfide) solution is 19.70 μA cm^−2^. The addition of [DEMEMA][FSI] to (3.5% NaCl + 10 ppm sulfide) solution yielded low corrosion current (*i*_corr_ values are 6.25, 3.43, 1.22 and 0.30 μA cm^−2^ for 50, 75, 100 and 120 ppm, respectively). Addition of [DEMEMA][FSI] to the corrosive solution resulted in a insignificant positive shifting in the *E*_corr_ (*E*_corr_ are − 247, − 229, − 220, − 211, − 203 mV for blank 50, 75, 100 and 120 ppm, respectively).

**Table 1:** Polarization parameters and corresponding inhibition efficiency for Cu–Ni alloy in (3.5% NaCl + 10 ppm sulfide) solution in the absence and presence of [DEMEMA][FSI] at 298 K.

[DEMEMA][FSI] conc (ppm)	*E*_corr_ mV (SCE)	*i*_corr_ μA cm^−2^	*B*_c_ (mV dec^−1^)	*B*_a_ (mV dec^−1^)	*E*_p_ %
Blank	− 247	19.70	− 123	138	–
50	− 229	6.25	− 93	83	68.2
75	− 220	3.43	− 74	74	82.5
100	− 211	1.22	− 65	66	93.8
120	− 203	0.30	− 45	56	98.4

The performance of [DEMEMA][FSI] as corrosion inhibitor (*E*_P_%) can be calculated by^33,34^:

2 $$E_{{\text{P}}} \% = \frac{{i_{{{\text{corr}}(0)}} - i_{{{\text{corr}}}} }}{{i_{{{\text{corr}}(0)}} }} \times 100$$

where *i*_corr(0)_ is the corrosion current density in blank solution.

The best performance of [DEMEMA][FSI] as corrosion inhibitor for Cu–Ni alloy in (3.5% NaCl + 10 ppm sulfide) solution was 98.4% at 120 ppm.

**Figure 2:**
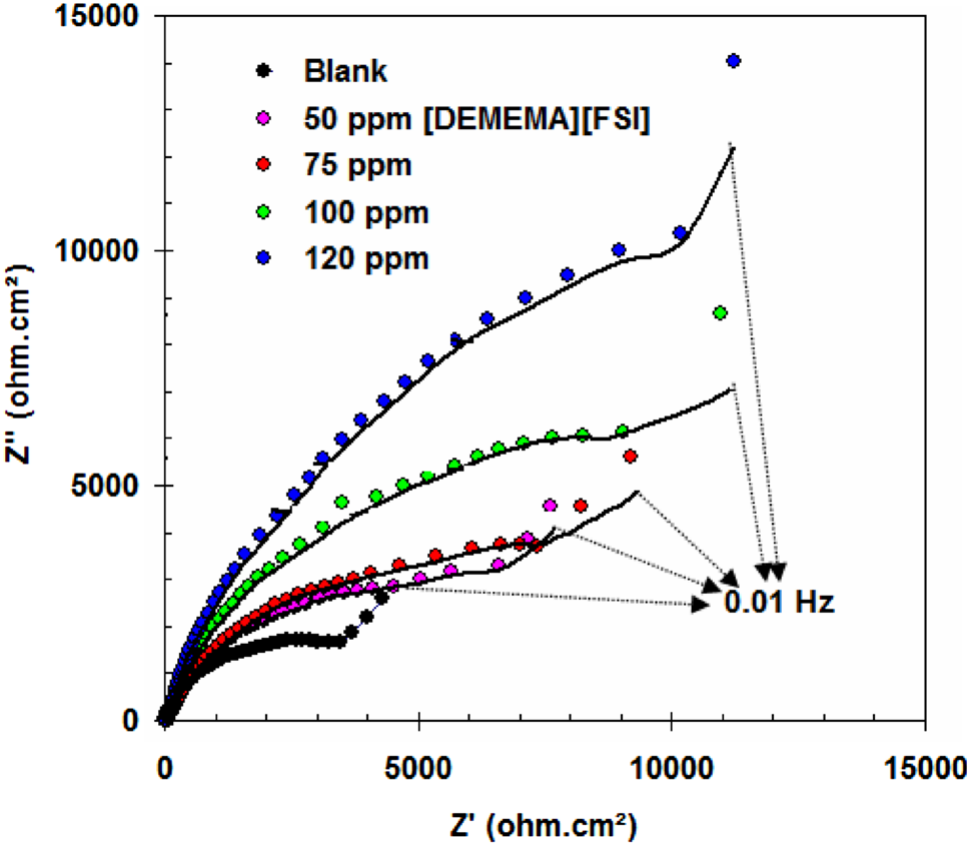
Nyquist plots of Cu–Ni alloy in (3.5% NaCl + 10 ppm sulfide) solution in the absence and presence of [DEMEMA][FSI] at 298 K.

EIS measurements were performed in (3.5% NaCl + 10 ppm sulfide) and in inhibited solutions. Typical EIS are presented in Fig. 2 in the form of Nyquist plots. All Nyquist plots display two depressed semicircle. The 1st one at high frequency zone and it is related to film layer^35,36^. The 2nd one at low frequency zone and it is related to corrosion reaction on the alloy surface^37,38^. The Nyquist plots exhibit Warburg impedance at low frequency zone due to diffusion process. The Warburg impedance seems to as a diagonal line with a 45° slope on a Nyquist plot. There is two phase maximum in all cases (Fig. 2). For (3.5% NaCl + 10 ppm sulfide) solution inhibited by [DEMEMA][FSI], the arc of Nyquist plots increase with DEMEMA][FSI] concentration (till 120 ppm) due to the increase in the surface coverage (*θ*) by [DEMEMA][FSI] molecules as shown in Table 2^39^.

**Table 2:** EIS parameters and corresponding inhibition efficiency for Cu–Ni alloy in (3.5% NaCl + 10 ppm sulfide) solution in the absence and presence of [DEMEMA][FSI] at 298 K.

Solution	Conc (ppm)	W (Ω cm^2^ s^−0.5^)	R_F_ (kΩ cm^2^)	Q_F_ (µF cm^-2^)	R_CT_ (kΩ cm^2^)	Q_dl_ (µF cm^-2^)	*θ*	E_R_ %
Blank	–	22.36	0.003	23.45	2.86	12.56	–	–
[DEMEMA][FSI]	50	12.59	0.005	18.10	7.87	10.88	0.6365	63.65
	75	10.63	0.009	12.90	10.45	8.76	0.7263	72.63
	100	4.93	0.015	11.77	17.79	7.09	0.8392	83.92
	120	4.45	0.017	4.95	32.99	5.32	0.9133	91.33

Surface coverage (*θ*) values were determined using impedance data, Eq. (3).

3 $$\theta = \, \left( {{\text{R}}_{{{\text{CT}}}} - {\text{ R}}^{0}_{{{\text{CT}}}} } \right) \, /{\text{ R}}_{{{\text{CT}}}}$$

R_CT_ and R^0^_CT_ represent the electron transfer resistance in inhibited and blank solutions, respectively.

**Figure 3:**
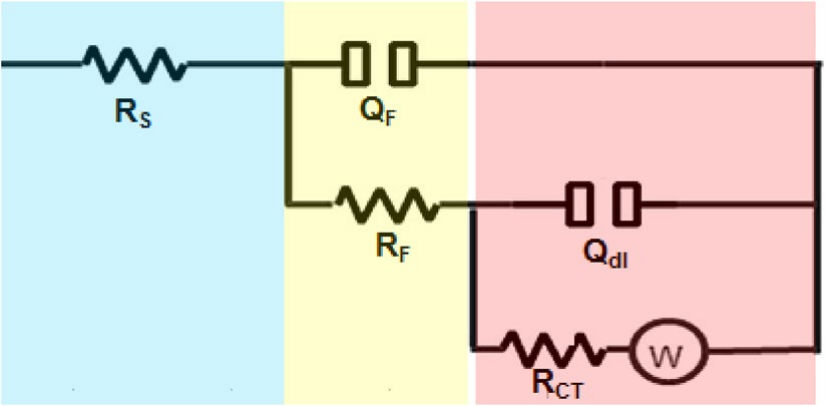
Equivalent circuit model used to fit the impedance measurement data.

The Nyquist plots can be well simulated using the equivalent circuit sketched in Fig. 3. Table 2 represents the EIS parameters obtained from Nyquist plots and equivalent circuit. In the case of [DEMEMA][FSI]-inhibited solutions, the charge transfer resistance (R_CT_) and film resistance **(**R_F_**)** increased, confirming the high corrosion resistance of the Cu–Ni alloy by the addition of [DEMEMA][FSI]. In addition, the decrease in the capacitance response of the double electrode and film layers (Q_dl_, Q_F_) by the addition of [DEMEMA][FSI] is mainly due to strong passive layer and/or the adsorbed [DEMEMA][FSI] molecules on the Cu–Ni alloy^40^. From the values of Warburg impedance (W) (see Table 2), it is evident that the corrosion of Cu–Ni alloy in (3.5% NaCl + 10 ppm sulfide) solution and in [DEMEMA][FSI]-inhibited solutions are mixed control by activation and diffusion^41^.

The values of charge transfer resistance in blank (R^0^_CT_) and inhibited solution (R_CT_) can be used to calculate the performance of [DEMEMA][FSI] as corrosion inhibitor (*E*_R_%) according to the following relation^42^:

4 $$E_{{\text{R}}} \% \, = \, \left[ { \, \left( {{\text{R}}_{{{\text{CT}}}} - {\text{ R}}^{0}_{{{\text{CT}}}} } \right) \, /{\text{ R}}_{{{\text{CT}}}} } \right] \, \times \, 100$$

From Table 2, the inhibition efficiency values of [DEMEMA][FSI] are in the range 63.65–91.33%. These values confirm the ability of [DEMEMA][FSI] to inhibit the corrosion Cu–Ni alloy in (3.5% NaCl + 10 ppm sulfide) solution.

### Gravimetric analysis

Table 3 displays the inhibition efficiency (*E*_W_%) and corrosion rate (*C*_R_) values obtained by gravimetric method at increasing doses of [DEMEMA][FSI] in (3.5% NaCl + 10 ppm sulphide) solution at 298 K.

**Table 3:** Gravimetric analysis for Cu–Ni alloy in (3.5% NaCl + 10 ppm sulfide) solution in the absence and presence of [DEMEMA][FSI] at 298 K.

[DEMEMA][FSI] conc (ppm)	*C*_R_ (μg cm^−2^ h^−1^)	*E*_w_ %
Blank	32.56	–
50	11.16	65.7
75	6.41	80.3
100	2.70	91.7
120	1.69	94.8

The *E*_W_% values were derived from the following relationship:

5 $$E_{{\text{R}}} \% \, = \, \left[ { \, \left( {C^{0}_{{\text{R}}} - C_{{\text{R}}} } \right) \, /C^{0}_{{\text{R}}} } \right] \, \times \, 100$$

*C*^0^_R_ and *C*_R_ represent the corrosion rate in the blank and inhibited solutions, respectively.

As [DEMEMA][FSI] is added to blank solution, the corrosion rate of Cu–Ni alloy in (3.5% NaCl + 10 ppm sulphide) solution decreases noticeably, and the inhibition efficiency increases with increasing [DEMEMA][FSI] concentration. At 120 ppm, the greatest inhibitory efficiency (94.8%) was achieved using gravimetric measurements. It's worth noting that the gravimetric data in Table 3 corroborate the EIS and polarisation data in Tables 1 and 2.

### Surface characterization

**Figure 4:**
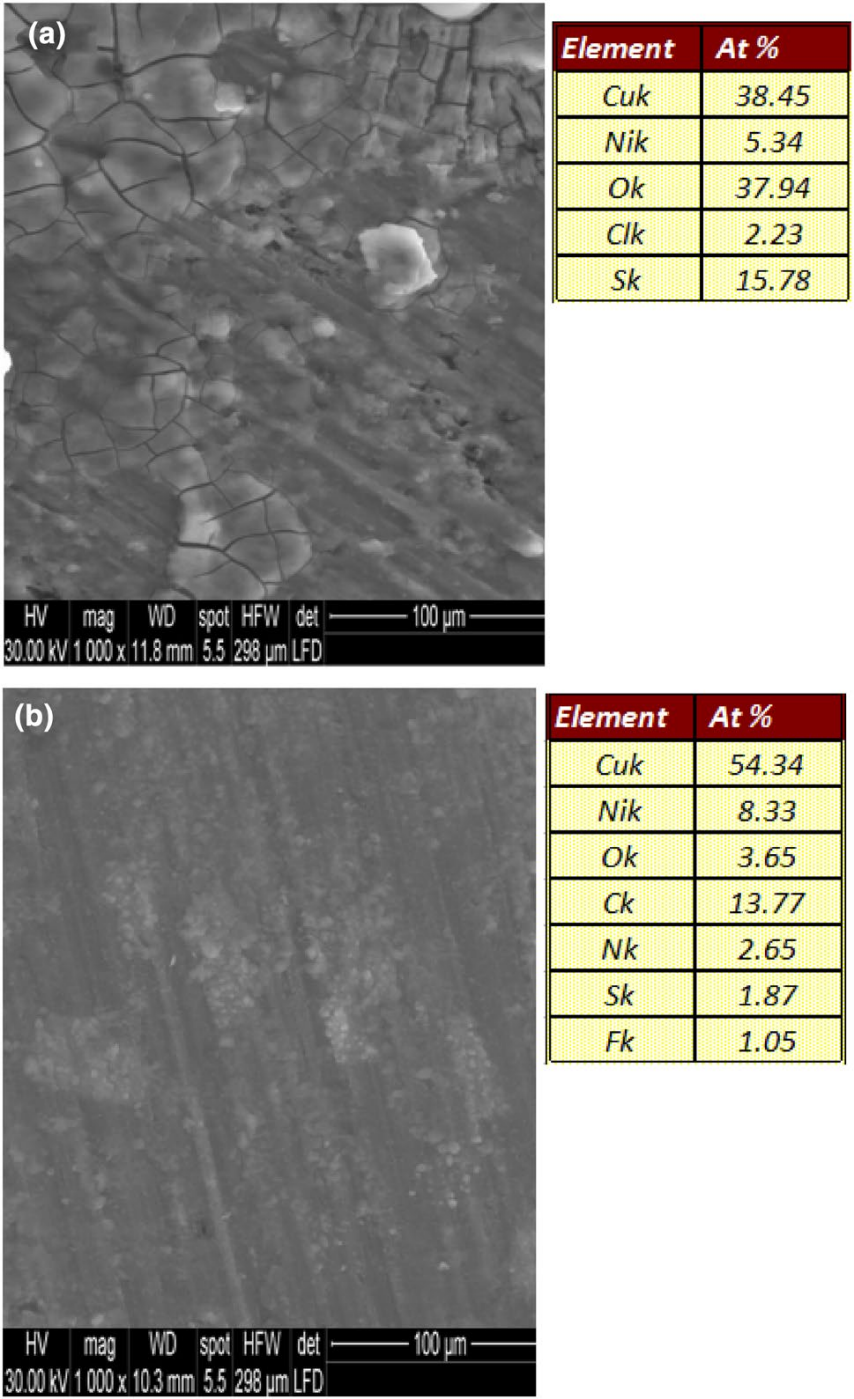
SEM and EDX images of the Cu–Ni alloy after 48 h immersion in (**a**) 3.5% NaCl + 10 ppm sulfide solution, (**b**) in 3.5% NaCl + 10 ppm sulfide + 120 ppm [DEMEMA][FSI] solution at 298 K.

In order to know some information on the composition of layer formed on the surface of Cu–Ni alloy in (3.5% NaCl + 10 ppm sulfide) solution in the absence and presence of [DEMEMA][FSI], the SEM and EDX analysis were performed and presented in Fig. 4. After Cu–Ni alloy immersion in (3.5% NaCl + 10 ppm sulfide) solution (Fig. 4a), the alloy surface was completely covered by black corrosion product and the surface suffered from severe crevices. According to the EDX analysis (recorded in Fig. 4a), the corrosion products are mainly copper oxide and copper sulfide. Furthermore, the image in Fig. 4b demonstrated a smooth alloy surface in the presence of [DEMEMA][FSI] with no noticeable change, indicating the high level of corrosion control provided by the [DEMEMA][FSI] to the surface of Cu–Ni alloy. The corresponding EDX analysis (recorded in Fig. 4b) reveals the presence of new signals C, N and F besides the very low intensity S and O signals. This confirms the adsorption of [DEMEMA][FSI] on the alloy surface. In addition, the Cl signal was not detected on alloy surface.

### Biological activities of SRB

The biological activities of SRB lead to sulfide ions generation in the seawater and this represents the major problem in the corrosion of Cu–Ni alloy in desalination plant^43^. In this part, the capacity of [DEMEMA][FSI] to inhibit the development of SRB in sea water will be investigated. Results obtained from SRB count in the 3.5% NaCl solution and biocidal efficiency (BE%) of [DEMEMA][FSI] are depicted in Table 4.

**Table 4:** Total SRB count and biocidal efficiency BE % of [DEMEMA][FSI].

[DEMEMA][FSI] conc (ppm)	SRB growth count CFU/ml	BE %
0.00	16.8 × 10^10^	–
50	10.5 × 10^9^	93.7
75	04.6 × 10^8^	99.7
100	00.4 × 10^2^	99.9
120	0	100

The BE% was evaluated from equation^44^:

6 $${\text{BE}}\% \, = \, \left[ {\left( {{\text{S}}^{0} - {\text{S}}} \right) \, /{\text{ S}}^{0} } \right] \, \times { 1}00$$

where S^0^ and S represent SRB count (CFU/ml) in 3.5% NaCl solution without and with [DEMEMA][FSI], respectively.

It can be seen that the addition of [DEMEMA][FSI] in the 3.5% NaCl solution can inhibit the growth of SRB even at low concentration. We found that the [DEMEMA][FSI] can completely eliminate the SRB from 3.5% NaCl solution (i.e. BE% = 100) at 120 ppm.

### Quantum chemical studies

**Figure 5:**
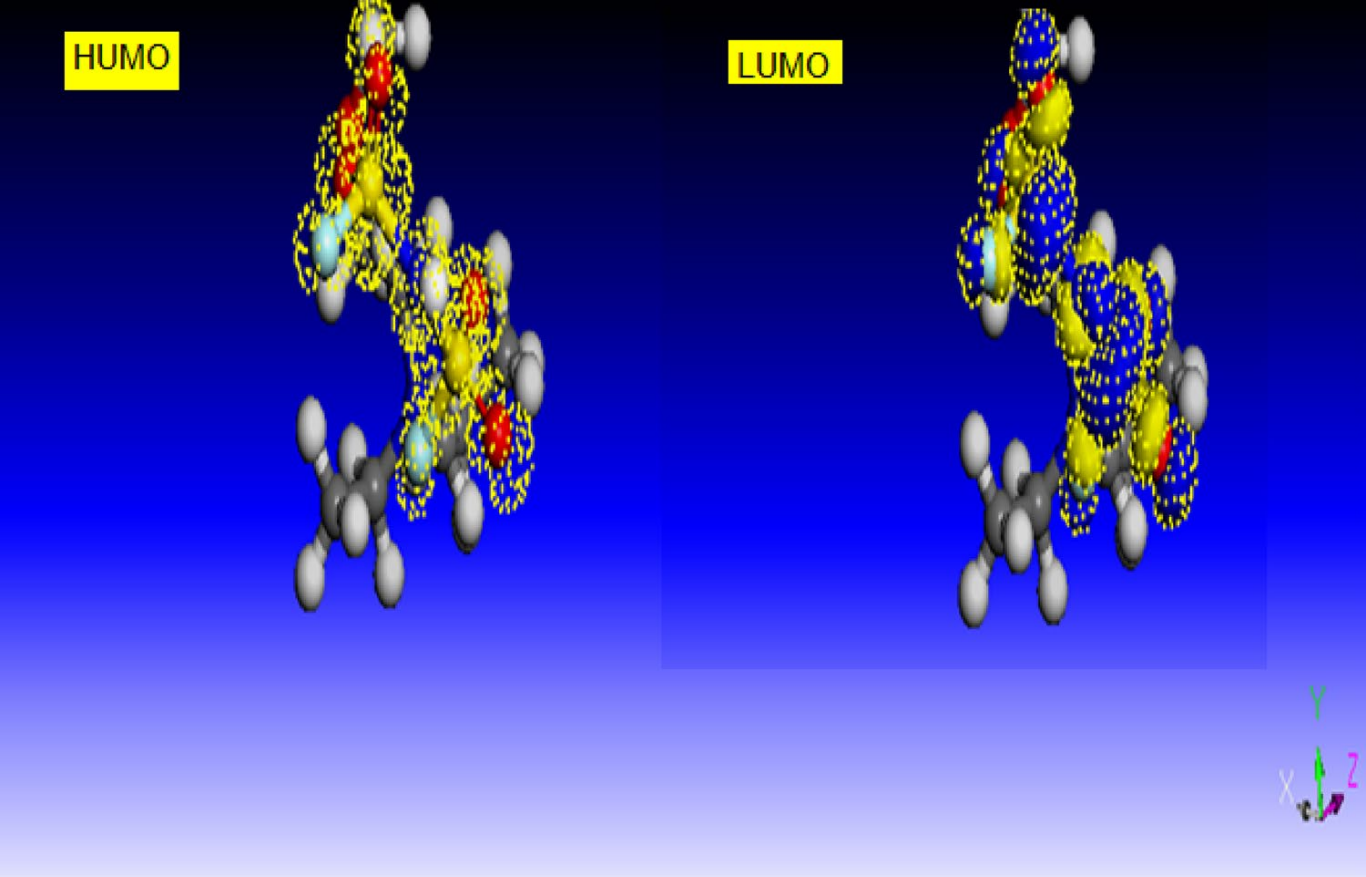
HOMO and LUMO of [DEMEMA][FSI] using B3LYP/6-31G (d,p). (H: white color; C: gray color; N: blue color and O: red color).

In this section, the theoretical prediction of the inhibition efficiency of [DEMEMA][FSI] was performed using Quantum calculations. The HOMO (highest occupied molecular orbital) and LUMO (lowest unoccupied molecular orbital) of [DEMEMA][FSI] are presented in Fig. 5. The charger distribution in the HOMO and LUMO is concentrated on the N, O and S atoms. This indicates that these atoms represent the adsorption centers^45^. The quantum calculations in Table 5 indicate that the [DEMEMA][FSI] has the high HOMO energy (i.e. *E*_HOMO =_  − 7.33 eV), which reflects the high ability of ionic liquid molecules to pay the electrons to the unoccupied orbital of Cu–Ni alloy^46^. On other hands, the low LUMO energy (i.e. *E*_LUMO =_ – 2.63 eV), reflects the high ability of ionic liquid molecules to receive the electrons from the Cu–Ni alloy. Moreover, the small energy band gap (i.e. Δ*E* = 4.7 eV) confirms that the interaction between [DEMEMA][FSI] molecules and Cu–Ni alloy surface is strong^47^. The dipole moment for [DEMEMA][FSI] is 0.48 debye. This indcates that [DEMEMA][FSI] molecules have high polarity leading to strong adsorption on the alloy surface^48^.

**Table 5:** Quantum chemical parameters of [DEMEMA][FSI].

*E*_HOMO_ eV	*E*_LUMO_ eV	Δ*E* eV	*Χ* eV	*η* eV	ΔN	Dipole moment (Deby)
− 7.33	–2.63	4.7	9.96	4.7	1.059	0.48

The following relationships are used to calculate the electronegativity (*χ*) and global hardness (η) variables for [DEMEMA][FSI]^49^:

7 $$\chi = \, 0.{5} \times ( - E_{{{\text{HOMO}}}} - E_{{{\text{LUMO}}}} )$$

8 $$h = \, 0.{5} \times ( - E_{{{\text{HOMO}}}} + E_{{{\text{LUMO}}}} )$$

A high *χ* value suggests a high chance to acquire electrons and, consequently, a high adsorption performance.

The number of electrons transfer from the molecule to the metal alloy (ΔN) is given by^49^:

9 $$\Delta {\text{N }} = \chi /{2}h$$

The value of ΔN (see Table 5) demonstrated an inhibition effect caused by transferring electrons, which is consistent with the findings of Lukovits et al.^50^. This finding confirmed the hypothesis that [DEMEMA][FSI] adsorption on the Cu–Ni alloy surface can occur as a result of donor–acceptor interactions between ionic liquid molecules and the alloy surface.

### Corrosion protection mechanism of [DEMEMA][FSI]

From the above results, we can conclude that [DEMEMA][FSI] molecules are able to protect the CuNi alloy from corrosion in sulfide containing 3.5% NaCl solution. The main strategy of the action of [DEMEMA][FSI] depends on two factors.

**Figure 6:**
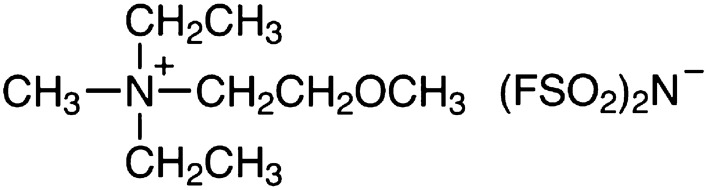
Molecular structure of (Diethyl (2-methoxyethyl)-methyl ammonium bis(fluorosulfonyl)imide) [DEMEMA][FSI].

The 1st factor is the adsorption of the [DEMEMA][FSI] molecules on the surface of CuNi alloy, leading to the isolation of the surface of the alloy from the corrosive saline water. In this case, the [DEMEMA][FSI] molecules are able to cover the cathodic and anodic sites. This because [DEMEMA][FSI] molecules have both negative and positive charges in their molecular structures (see Fig. 6).

The adsorption processes can be summarized in the following steps^51^:

1. At anodic sites
  10 $${\text{CuNi }} + {\text{ nH}}_{{2}} {\text{O }} \to \left[ {{\text{CuNi}}} \right]\left( {{\text{H}}_{{2}} {\text{O}}} \right){\text{n}}_{{{\text{ads}}}}$$
  11 $$\left[ {{\text{CuNi}}} \right]\left( {{\text{H}}_{{2}} {\text{O}}} \right){\text{n}}_{{{\text{ads}}}} + {\text{ nCl}}^{ - } \left( {{\text{from}}\;{\text{ saline }}\;{\text{water}}} \right) \, \to \, \left( {{\text{CuNi}}} \right) \ldots \left[ {\left( {{\text{H}}_{{2}} {\text{O}}} \right){\text{n}}_{{{\text{ads}}}} \ldots {\text{nCl}}} \right]^{ - }_{{{\text{ads}}}}$$
  12 $$\left( {{\text{CuNi}}} \right) \ldots \left[ {\left( {{\text{H}}_{{2}} {\text{O}}} \right){\text{n}}_{{{\text{ads}}}} \ldots {\text{nCl}}} \right]^{ - }_{{{\text{ads}}}} + \, \left[ {{\text{DEMEMA}}} \right]^{ + } \to \, \left( {{\text{CuNi}}} \right) \ldots \left[ {\left( {{\text{H}}_{{2}} {\text{O}}} \right){\text{n}}_{{{\text{ads}}}} \ldots {\text{nCl}}} \right]^{ - } \left[ {{\text{DEMEMA}}} \right]^{ + }$$
  13 $$\left( {{\text{CuNi}}} \right) \ldots \left[ {\left( {{\text{H}}_{{2}} {\text{O}}} \right){\text{n}}_{{{\text{ads}}}} \ldots {\text{Cl}}} \right]^{ - } \left[ {{\text{DEMEMA}}} \right]^{ + } \to \left( {{\text{CuNi}}} \right)\left[ {{\text{DEMEMA}}} \right]_{{{\text{ads}}}} + {\text{ nH}}_{{2}} {\text{O }} + {\text{ nCl}}^{ - }$$
  and/or
  14 $${\text{CuNi}} + \, \left[ {{\text{FSI}}} \right]^{ - } \to {\text{ CuNi}}\left[ {{\text{FSI}}} \right]^{ - }_{{{\text{ads}}}}$$
  15 $${\text{CuNi}}\left[ {{\text{FSI}}} \right]^{ - }_{{{\text{ads}}}} + \left[ {{\text{DEMEMA}}} \right]^{ + } \to {\text{ CuNi}}\left[ {{\text{FSI}}} \right]^{ - } \left[ {{\text{DEMEMA}}} \right]^{ + }_{{{\text{ads}}}}$$
2. At cathodic sites
  16 $${\text{CuNi}}\left( {\text{cathodic sites}} \right) \, + \, \left[ {{\text{DEMEMA}}} \right]^{ + } + {\text{ e }} \to {\text{ CuNi }}\left( {\text{cathodic sites}} \right)\left[ {{\text{DEMEMA}}} \right]_{{{\text{ads}}}}$$

The adsorption of [DEMEMA][FSI] on the CuNi alloy occurs through heteroatoms N, O and S atoms^52–55^.

The 2nd factor is the ability of [DEMEMA][FSI] to inhibit the sulfide ions generation in the seawater by de-activating SRB. The biocidal efficiency of [DEMEMA][FSI] is due to the adsorption of cationic part of ionic liquid (i.e. [DEMEMA]^+^) on the SRB cell wall and followed by penetration inside the cell^56,57^.This leads to the degradation of proteins and nucleic acids inside the SRB cell.

## Conclusions

In this study, for the first time, ammonium based ionic liquid (Diethyl (2-methoxyethyl)-methyl ammonium Bis(fluorosulfonyl)imide) [DEMEMA][FSI] was used to protect CuNi alloy from corrosion in sulfide containing seawater medium. The role of [DEMEMA][FSI] depends on the decrease of the corrosive action of seawater and the inhibition of the production of sulfide ions from biological activities of SRB. The electrochemical (polarization and EIS) and Gravimetric examination revealed the inhibitive effects of [DEMEMA][FSI] on CuNi alloy corrosion in (3.5% NaCl + 10 ppm sulfide) solution with efficiency reaches to 98.4% at 120 ppm. In the case of [DEMEMA][FSI]-inhibited solutions, the charge transfer resistance and film resistance increased, confirming the high corrosion resistance of the Cu–Ni alloy by the addition of [DEMEMA][FSI]. Further, the use of DEMEMA][FSI] was found to be very effective for deactivating the growth of SRB in seawater. Surface screening studies by SEM/EDX, prove the presence of DEMEMA][FSI] film on Cu–Ni alloy surface, confirming the adsorption process. Theoretical quantum studies were used to verify experimental findings. This study will aid in maximizing the desalination plant lifetime, lowering the cost of corrosion and providing low environmental problems.
